# Acceleration and suppression of resistance development by antibiotic combinations

**DOI:** 10.1186/s12864-017-3718-2

**Published:** 2017-04-26

**Authors:** Shingo Suzuki, Takaaki Horinouchi, Chikara Furusawa

**Affiliations:** 1 Laboratory for Multiscale Biosystem Dynamics, Quantitative Biology Center (QBiC), RIKEN, 6-2-3 Furuedai, Suita, Osaka 565-0874 Japan; 2 Universal Biology Institute, The University of Tokyo, 7-3-1 Hongo, Bunkyo-ku, Tokyo 113-0033 Japan

**Keywords:** Laboratory evolution, *Escherichia coli*, Antibiotic resistance

## Abstract

**Background:**

The emergence and spread of antibiotic resistance in bacteria is becoming a global public health problem. Combination therapy, i.e., the simultaneous use of multiple antibiotics, is used for long-term treatment to suppress the emergence of resistant strains. However, the effect of the combinatorial use of multiple drugs on the development of resistance remains elusive, especially in a quantitative assessment.

**Results:**

To understand the evolutionary dynamics under combination therapy, we performed laboratory evolution of *Escherichia coli* under simultaneous addition of two-drug combinations. We demonstrated that simultaneous addition of a certain combinations of two drugs with collateral sensitivity to each other could suppress the acquisition of resistance to both drugs. Furthermore, we found that the combinatorial use of enoxacin, a DNA replication inhibitor, with Chloramphenicol can accelerate acquisition of resistance to Chloramphenicol. Genome resequencing analyses of the evolved strains suggested that the acceleration of resistance acquisition was caused by an increase of mutation frequency when enoxacin was added.

**Conclusions:**

Integration of laboratory evolution and whole-genome sequencing enabled us to characterize the development of resistance in bacteria under combination therapy. These results provide a basis for rational selection of antibiotic combinations that suppress resistance development effectively.

**Electronic supplementary material:**

The online version of this article (doi:10.1186/s12864-017-3718-2) contains supplementary material, which is available to authorized users.

## Background

The emergence of antibiotic-resistant bacteria is a serious and worsening global public health problem, despite our efforts to suppress and to control it [1, 2]. Antibiotic resistance is achieved through genetic changes, either by acquisition of resistance genes through horizontal gene transfer or by *de novo* mutations. Clinical uses of antibiotics have provided a selective advantage for naturally emerged resistant bacteria which renders antibiotics ineffective [3]. Although chemically altered or newly discovered compounds have been developed to combat such antibiotic resistant bacteria [4], an emergence of antibiotic resistance always follows the clinical introduction of new antibiotics [5]. Furthermore, the discovery and development of new antibiotics have stagnated recently [6], and thus we need to develop ways to suppress such emergence of antibiotic resistant strains by using the currently available repertoire of antibiotics and other chemical substrates [7].

The use of multidrug combinations, both sequentially and simultaneously, is increasingly important to counteract the emergence of antibiotic resistant bacterial pathogens [8]. The effects of the simultaneous use of multiple drugs have been extensively studied. For example, a network of drug-drug interactions including additive, antagonistic, and synergistic interactions was quantified, which potentially affect the likelihood of the emergence of resistance [9, 10]. Several studies demonstrated that acquisition of resistance to one antibiotic is accompanied with changes of resistance levels to various drugs, called collateral resistance and sensitivity, which also significantly contribute to determining the course of antibiotic resistance evolution [11–14]. A better understanding of these phenomena is important since they could potentially inform about strategies to suppress the emergence of resistant strains by using multidrug combinations. However, the number of quantitative studies about combinatorial use of multiple drugs was limited [15–18], despite its importance for medical applications.

In this study, we performed laboratory evolution of *Escherichia coli* under antibiotic combinations to quantify how the change of phenotype and genotype is affected by the simultaneous use of multiple drugs. For each evolved strain under single or combinatorial use of antibiotics, genome sequence analysis was carried out to identify fixed mutations. Furthermore, we evaluated the effect of fixed mutations on antibiotic resistance by reconstructing mutants by introducing a selection of the identified mutations. In addition, our data suggested that the simultaneous use of quinolone and other types of antibiotics can accelerate the acquisition of resistance in comparison with the single use of either drug. We also demonstrated that the simultaneous use of drugs showing collateral sensitivity to each other can suppress the acquisition of resistance to both drugs.

## Results and discussion

### Procedure of laboratory evolution of antibiotic resistance

**Fig. 1:**
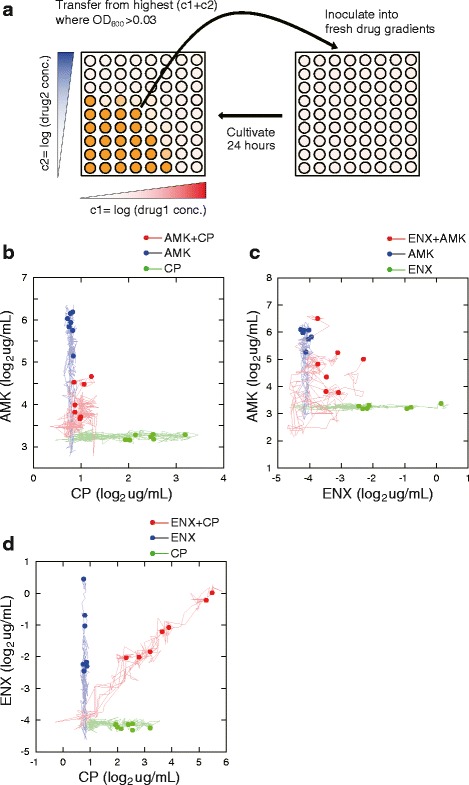
Laboratory evolution under a combination of two antibiotics. **a** The procedure of laboratory evolution. The laboratory evolution was performed in 8 × 8 wells in 96-well microplates with two antibiotic gradients. The dilution step was set at 2^0.25^ fold. At a daily transfer, the bacterial cells were transferred from the well with the highest sum of log-transformed drug concentrations in which cells were able to grow (OD_600_ > 0.03). **b**-**d** The change of MIC in laboratory evolution. The trajectories of MIC changes are plotted for **b** AMK + CP, **c** AMK + ENX, and **d** CP + ENX, respectively. The *dots* represent MICs at the end-point of laboratory evolution (33 days), and the *lines* show the changes of MIC during laboratory evolution. The *red dots* and *lines* correspond to the trajectories of evolution using combinatorial use of two antibiotics, while *blue and green dots/lines* show the results of laboratory evolution under addition of single antibiotics. For the single drug experiments, MICs to the counterpart drug were assumed to be identical to the parent strain in these figures. The data points have been slightly randomized by adding Gaussian noise to avoid overlapping of points

To explore effects of combinatorial use of antibiotics on the resistance evolution, we selected 3 antibiotics, i.e., amikacin (AMK; aminoglycoside type protein synthesis inhibitor), chloramphenicol (CP; bacteriostatic protein synthesis inhibitor), and enoxacin (ENX; quinolone-type DNA replication inhibitor). We used these drugs for the following two reasons. First, we confirmed that resistance to these antibiotics can be acquired by *de novo* mutations by laboratory evolution, as shown in our previous study [11]. Second, the same study demonstrated that the resistant strains to these drugs exhibited collateral resistance/sensitivity to other drugs, which likely contribute to determining the course of antibiotic resistance evolution. We performed laboratory evolution of *E. coli* in the presence of 3 pairwise combinations of above antibiotics and 3 single antibiotics, totaling 6 conditions. In the laboratory evolution with drug combinations, *E. coli* cells were cultured in a synthetic medium with serial dilutions of antibiotics in two-dimensional arrays of 8 × 8 wells, as shown in Fig. 1a. Every 24 h, cells were propagated from a well with the largest product of drug concentrations in which cells were able to sustain growth (see Methods for details). This experimental setting enabled us to impose a selection pressure for developing resistance to both drugs simultaneously.

In addition to the laboratory evolution under combinatorial use of two antibiotics, we performed laboratory evolution under addition of single antibiotics, as control experiments, i.e., under the addition of AMK, CP, or ENX. In these laboratory evolutions, cells were cultured in media with 8 different concentrations of drugs and were propagated daily from a well containing the highest drug concentration in which the cells were able to sustain growth. To evaluate the reproducibility of the evolution, both for the cases of combinatorial and single use of antibiotics, 7 independent culture lines were propagated in parallel.

### Result of laboratory evolution under combinatorial use of antibiotics

**Fig. 2:**
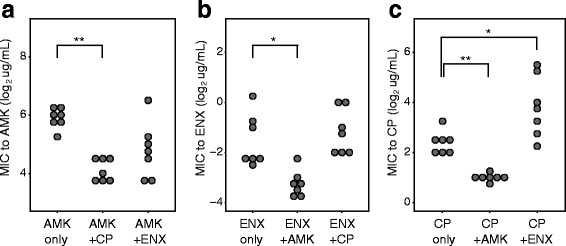
MICs of evolved strains. The MICs of evolved strains to (**a**) amikacin (AMK), **b** enoxacin (ENX), **c** chloramphenicol (CP) are presented. For each end-point culture of the laboratory evolution, we isolated a clone and quantified MICs of it. The symbols * and ** are *p*-values (*p* < 0.05, *p* < 0.001, respectively) obtained by Steel-Dwass test

Figure 1b to d show the change in minimum inhibitory concentrations (MIC) during 33 days propagation. For the results with the combinatorial use of two drugs (red symbols in Fig. 1b to d), the changes of MIC to both drug are plotted on a 2-dimensional plane. Here, we defined MIC as the concentration of drug from which cells were propagated. For the results under single drug addition that are overlaid in these figures, we assume that MICs for the counterpart drug do not change during evolution. Figure 2a to c present MICs of the isolated evolved strains.

As shown in Figs. 1b and 2, the combinational use of AMK and CP significantly suppressed the increase in MICs for both AMK (Fig. 2a) and CP (Fig. 2c) in comparison with their single use. Several previous studies reported that *E. coli* cells show a mutual collateral sensitivity between CP and aminoglycoside drugs including AMK, that is, when *E. coli* cells acquire resistance to CP, the cells become more sensitive to AMK than the parent strain, and vice versa [11–13]. Also in this study, some of resistant strains obtained under sole use of AMK and CP exhibited a collateral sensitivity as shown in Additional file 1: Figure S1. Thus, the result strongly suggested that the observed suppression of resistance development by the combinatorial use of CP and AMK was caused by the trade-off nature of CP and AMK resistance, which was consistent with previous studies [16, 19].

For the combinatorial use of AMK and ENX, we also found a suppression of resistance development (Fig. 1c) in comparison with the single use of drugs, although it was smaller than the case of AMK and CP. The change of MIC to ENX was significantly suppressed by the combinatorial use of AMK and ENX (Fig. 2b), while the effect was not statistically significant for the AMK resistance (Fig. 2a). Some resistant strains obtained under the single use of AMK and ENX showed collateral sensitivity as shown in Additional file 1: Figure S1. Thus, these data might also support that the combinatorial use of two drugs showing collateral sensitivity suppresses the development of resistance.

Interestingly, the combinatorial use of CP and ENX exhibited an acceleration of acquisition of resistance to CP (Figs. 1d and 2c). In contrast, the changes of MIC to ENX were almost identical between the combinatorial use and the single use of ENX. Previous studies suggested that the effect of combinatorial addition of CP and DNA gyrase inhibitor is antagonistic [9]. Thus our result of acceleration of resistance development is inconsistent with the previous studies showing that drug combinations with antagonistic interactions suppress the development of resistance [10, 20, 21]. Our previous data demonstrated that the combination of CP and ENX has mutual collateral resistance, i.e., when *E. coli* cells acquire CP resistance, the cells simultaneously acquire ENX resistance, and vice versa [11]. Also in this study, the resistant strains obtained by the single use of CP and ENX exhibited collateral resistance, as shown in Additional file 1: Figure S1. However, the observed collateral resistance was symmetric, and thus it is difficult to explain the asymmetric acceleration of resistance acquisition at this point. One possibility to explain the asymmetric acceleration is a change of mutation fixation rate by addition of antibiotics. Therefore, in the following parts, we present the analysis of genomic sequences in the obtained resistant strains.

### Mutations fixed in evolved strains

**Fig. 3:**
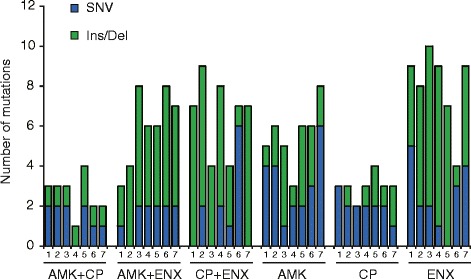
The number of fixed mutations in evolved strains. Mutations were identified using Illumina MiSeq system (see Methods for details). *Blue* and *green bars* represent single nucleotide variation (SNV) and ins/del, respectively

To analyze the contribution of genetic mutations to the acquisition of resistances, we isolated a single clone at the end-point of each culture, and then performed whole-genome resequencing analysis of 42 evolved clones (6 conditions × 7 independent culture series). Figure 3 shows the number of mutations identified in the evolved strains. Detailed information about the mutations is presented in Additional file 2: Table S1. Ten or fewer mutations were fixed in each of the evolved strains. An important point here is that the numbers of fixed mutations in the strains evolved under addition of ENX (i.e., AMK and ENX, CP and ENX, and ENX only conditions) were larger than those without the addition of ENX (Steel-Dwass test, *p* < 10^−5^). It is known that quinolones induce the SOS mutagenic response, in particular, Ins/Del mutagenesis [22–26], and in fact the numbers of Ins/Del mutations fixed in the ENX exposed strains were significantly larger than that of the latter (Steel-Dwass test, *p* < 10^−6^).

Among these identified mutations, we found several characteristic mutations that are commonly fixed in resistant strains obtained by specific combinations of drugs. For example, multiple resistant strains obtained under the addition of AMK and CP had mutations in *rsxC*, *rsxD*, *ybeX*, and *yojL* genes, while no resistant strain obtained by the single use of drugs had mutations in these genes. These results might suggest that the above mutations contributed to the simultaneous acquisition of resistance to AMK and CP, although the increases in resistance were small as shown in Fig. 1b. *rsxC* and *rsxD* genes are involved in a reducing system of the superoxide sensor SoxR, whose disruption causes constitutive expression of SoxS [27]. Since overexpression of SoxS is known to cause multiple antibiotic resistances [28], the identified mutations *rsxC*/*rsxD* genes might contribute AMK and CP resistance via regulation of SoxS. *yojL* encodes a flavin transferase catalyzes the transfer of flavin mononucleotide to target proteins [29]. *ybeX* encodes a putative membrane protein predicted to be involved in Mg^2+^ and Co^2+^ efflux [30]. There is no report so far on the relationship between functions of above genes and antibiotic resistance, and the mechanism how these genes contribute to the resistance to AMK and CP is unclear. In addition, 3 out of 7 resistant strains obtained by the simultaneous addition of AMK and ENX had mutations in *cyaA* gene, which encodes adenylate cyclase involved in the cyclic AMP synthesis and metabolic control [31]. It was suggested that disruption of *cyaA* gene is involved in the emergence of quiescent bacteria called persisters which can survive antibiotic treatment [32]. The detailed mechanism how *cyaA* mutations contribute to the simultaneous acquisition of AMK and ENX remains unclear.

### Analysis of mutation effects by constructing mutant strains

The whole genome resequencing analysis indicated that the number of fixed mutations in the ENX exposed strain was significantly larger. By assuming similarity in the selection strength, the number of available mutations causing resistance acquisition, and the effective population size among the laboratory evolutions, this result might suggest an increase in mutation rate by the ENX addition, as demonstrated by the previous study using similar antibiotics [24]. If this is the case, one possible explanation for the observed acceleration of CP resistance development by the combinatorial use of CP and ENX is due to the increased mutation rate with ENX addition. The data demonstrated that a majority of fixed mutations in evolved strains under CP and ENX addition were Ins/Del, and almost all of short Ins/Del (25 out of 26 Ins/Del; less than 100 bp) were accompanied by a frame shift. Such loss of function mutations by Ins/Del might contribute to the CP and ENX resistance. Another possible explanation for the acceleration of resistance evolution is epistasis between fixed mutations [33]. For example, when a mutation responsible for ENX resistance has positive epistasis with mutations which caused CP resistance, such interaction might accelerate the resistance evolution. To quantitatively evaluate the effect of mutations and the possible contribution of epistasis to the acceleration of resistance development, we constructed deletion mutants of genes in which mutations were commonly fixed in the evolved strains under combinatorial use of CP and ENX, i.e., *acrR*, *marR*, *ompF*, and *glyXY*. Since the mutations fixed in these genes were Ins/Del which caused frame-shifts, we evaluated the effect of these mutations on the resistance by deleting open reading frames. Also, we found that multiple mutations were fixed in genes in the *atp* operon which caused frame-shifts, which suggested that disruption of these genes encoding subunits of the ATP synthase complex contributed to CP and/or ENX resistance. To evaluate this possibility, we also constructed a deletion strain of *atpI*, the first gene in the *atp* operon, whose deletion is known to decrease ATPase activity [34].

**Fig. 4:**
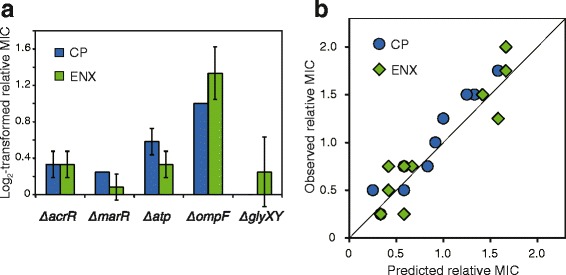
Changes in relative MICs by deletion of genes. **a** The log_2_-transformed relative MICs (CP and ENX) to the parent strain observed in *acrR*, marR, *atpI*, *ompF*, and *glyXY* gene deletion mutants. The error bars represent the standard deviation of MICs obtained from 3 independent cultures. The bar for the relative MIC of glyXY deletion strain to CP is invisible, since the value of log2-transformed relative MIC is zero with zero standard deviation. **b** Predicted and observed relative MICs (CP and ENX) in double deletion mutants. Double deletion mutants were constructed, in which all possible pairwise combinations of above 5 genes were disrupted. The predicted MICs in double deletion mutants were calculated by a sum of log_2_-transformed relative MICs of corresponding two single-gene deletion mutants. The *solid black line* represents y = x for reference

Figure 4a shows CP and ENX resistances of the deletion mutants quantified by MIC relative to the parent strain. As shown, CP and ENX resistances increased by the gene deletions, except for *glyXY* gene deletion. AcrR encoded in *acrR* has been known to repress the expression of the multi-drug efflux pump (AcrAB) and deletion of *acrR* activates the expression of the multi-drug efflux pump [35]. OmpF is an outer membrane porin, which is a non-specific transport channel that allows for the passive diffusion of antibiotics. It is known that a decrease in *ompF* expression results in decreasing antibiotic uptake, which leads to resistance [36]. In addition to these well-known genes which contribute to the antibiotic resistances, we confirmed that the deletion of *atpI* also increases CP and ENX resistance. It is known that ATP synthesis by ATP synthase is driven by proton motive force (PMF). Note that the multi-drug efflux pump (AcrAB) is a proton antiporter and also driven by the PMF [37, 38], which indicates that the activity of the multi-drug efflux pump competes with ATP synthesis in the cell membrane. Thus, the disruption of ATP synthesis can facilitate multi-drug efflux activity, leading to the resistances to some types of drugs. Our result of resistance acquisition in *atpI* deletion mutant supports this hypothesis. *marR* encodes a regulator protein involved in multiple antibiotic resistance [39]. It was demonstrated that the disruption of *marR* causes acquisition of resistance to various drugs through activation of AcrAB multi-drug efflux pump [40]. Our result of resistance acquisition in *marR* deletion mutant was consistent with the previous study. *glyXY* encodes glycine tRNA, and the relationship between antibiotic resistance and disruption of glycine tRNA has not been reported.

### Analysis of epistatic interactions between mutations

To evaluate the epistatic effect between these mutations, we constructed double deletion mutants of all possible pairwise combinations of these five mutations (10 combinations in total), and quantified the CP and ENX resistance of these double deletion mutants. By comparing the resistance of single and double deletion mutant strains, we analyzed whether the resistance of double deletion mutants can be explained by a sum of the effect in corresponding single deletion mutants, or if non-additive interactions between deletions exist. Figure 4b shows the relationship between the log-transformed relative MIC of the double deletion mutant and the sum of log-transformed relative MICs of corresponding single deletion mutant. The data points are close to the diagonal line (the coefficient of determination was 0.87), which suggested that the epistatic interactions between these gene deletions are negligible. This result supported the hypothesis that the observed acceleration of CP resistance development by combinatorial use of CP and ENX was caused by an increase of mutation rate by the addition of ENX.

There are, of course, other possibilities to explain the observed acceleration of CP resistance development. One possibility is due to the collateral resistance between CP and ENX [11], which could influence the selective advantage and the number of possible mutations contributing the resistance, to increase the fixation rate of resistant mutations. Another possibility is due to higher-order epistasis among resistant mutations [41], which cannot be analyzed by the pairwise combination of identified mutations. Furthermore, in this study we investigated the effects of only a part of identified mutations on the acquisition of resistance. Therefore, we could not exclude the possibility that other mutations which were not tested contributed to the observed resistance acquisition and unknown epistatic interactions. Further research to evaluate effects of such infrequent mutations is necessary, for complete understanding of the evolutionary dynamics under the combinatorial drug uses. For this purpose, the use of high-throughput genome engineering such as MAGE method [42] is desirable.

The result that the combinatorial use of AMK and ENX did not accelerate AMK resistance development (Fig. 1c) seems inconsistent with the suggested increase of mutation fixation frequency by the ENX addition as discussed above. One possible explanation for this result is due to collateral sensitivity between AMK and ENX, i.e., acquisition of ENX resistance causes AMK sensitivity, which was presented in our previous study [11]. Also in this study, the data of ENX resistant strains obtained by the single use of ENX exhibited collateral sensitivity to AMK (Additional file 1: Figure S1). As shown in Fig. 1c, the resistance to ENX increased by the combinatorial use of ENX and AMK in comparison to the parent strain. This resistance to ENX can cause sensitivity to AMK due to collateral sensitivity, which might result the suppression of acquisition of resistance to AMK.

## Conclusions

In this study, we performed laboratory evolution of *E. coli* under combinatorial use of antibiotics. We demonstrated that addition of ENX accelerated the development of CP resistance compared to the single use of CP. We also found that resistance acquisition was significantly suppressed by the combinatorial use of antibiotics exhibiting collateral sensitivity each other. Whole-genome sequencing showed that the addition of ENX significantly increased Ins/Del mutations which might suggest the contribution of increased mutation rate to the acceleration of CP resistance acquisition. Although further research including clinical studies is required, these results indicated that selection of antibiotics is very important for suppression of antibiotic resistance acquisition in combination therapy. Further studies to unveil the complex interactions among antibiotic resistances are important for designing rational ways to control the development of antibiotic resistance.

## Methods

### Growth medium, bacterial strain, and antibiotics

All experiments were performed in modified M9 medium [43].The IS elements-free *Escherichia coli* strain MDS42 [44] was purchased from Scarab Genomics and utilized throughout this study. The use of the IS elements-free strain can make the resequencing analysis reliable since the determination of the precise position of IS element insertions is often difficult by using high-throughput sequencers. All antibiotics used in this study were purchased from Wako Pure Chemical Industries, Ltd. Antibiotic stock solutions were made by dissolving powder stocks in specified solvents by manufacture’s instruction. All antibiotic stocks dissolved in water were 0.2 μm filter-sterilized. The antibiotic stock solutions were stored at −80 °C prior to use.

### Laboratory evolution

The bacterial cells were cultured in 200 μl modified M9 medium in a 96-well microplate (Corning Inc. 3595) with shaking at 900 strokes min^−1^ on a microplate shaker TITRAMAX1000 (Heidolph Instruments) at 34 °C. We used the incubation temperature of 34 °C instead of the commonly used temperature (37 °C) to shorten the time that the cells are in the stationary phase and the death phase. For laboratory evolution under a single drug, the bacterial cells were propagated in modified M9 medium with 16 different concentrations in 2^0.25^ fold dilution steps of antibiotics. For laboratory evolution under combinatorial use of antibiotics, antibiotics were mixed in a two-dimensional array of 8 × 8 wells in which the concentration of each antibiotic varied in 2^0.25^ fold dilution steps of antibiotics. Antibiotic serial diluted plates were prepared using a Zephyr Compact Liquid Handling Workstation (PerkinElmer Inc.). At a daily transfer, the growth of the cells was monitored by measuring the OD600 nm of each well using the microplate reader 1420 ARVO (PerkinElmer Inc.). We defined a well whose OD600 nm was greater than 0.03 as the well in which cells could grow, which corresponds to the mid-log phase in our culture system. For laboratory evolution under a single drug, the cells, calculated to yield an initial OD600 nm of 3 × 10^−5^ corresponding to approximately 10^3^ cells per well, were transferred from the well with highest drug concentrations in which cells could grow to freshly prepared plates with serial diluted antibiotic. For laboratory evolution under combinatorial use of antibiotics, the bacterial cells were transferred from the well with the highest sum of log-transformed concentrations of the two antibiotics which permitted cellular growth. When there were multiple wells with an identical product of drug concentrations, we selected a well with highest cell concentration (OD600 nm value) from these wells. The cells during the evolution experiments were stored as glycerol stocks at -80 °C and used for further analysis. To evaluate the reproducibility of the evolution of resistance, for each condition, 7 independent culture lines were propagated in parallel. To isolate clonal cell populations, evolved cells from frozen glycerol stocks were plated on modified M9 agar plate and incubated at 34 °C for 2 days. Three to six colonies were picked and suspended in modified M9 medium. The suspensions were stored at −80 °C with glycerol.

### MIC measurement

Serial dilutions of antibiotics were made in 96-well microplates using modified M9 medium by the liquid handling workstation and stored at −80 °C prior to use. The ranges of antibiotic concentrations used for determining MICs were in 2^0.25^ fold dilution steps. We prepared precultures by shaking glycerol stocked strains in 200 μL of modified M9 medium in 96-well microplates for 23 h at 34 °C without antibiotics. The cells in the preculture, calculated to yield an initial OD600 nm of 3 × 10^−5^, were inoculated into each well in freshly thawed plates with the drug gradients to a final volume 200 μL. After 23 h incubation with shaking, the microplates were read at 600 nm using the microplate reader. The MICs were defined as the lowest concentration of antibiotics that reduced the growth to an OD600 nm of less than 0.03.

### Genomic DNA preparation

To extract and purify genomic DNA of each clone, we prepared precultures by shaking stocked strains in 200 μL of modified M9 medium in 96-well microplates for 23 h at 34 °C without antibiotic. The cells precultured were diluted to OD600 nm of 3 × 10^−5^ into 10 mL of fresh modified M9 medium in test tubes without antibiotic again. Cell culture was performed at 34 °C for 23 h with shaking at 150 strokes min^−1^ using water bath shakers Personal-11 (Taitec Co.). After the transfer of 200 μL of cultures to a microplate, we confirmed that cultures grown in test tubes reached OD600 nm values of more than 0.2. Rifampicin (final concentration 300 μg/mL) was subsequently added, and culture was continued for a further 3 h to block initiation of DNA replication. We used rifampicin here to eliminate partially replicated genomes in the sample since they can cause a bias in the coverage depth. The cells were collected by centrifugation at 25 °C at 20,000 × g for 5 min, and the pelleted cells were stored at −80 °C prior to genomic DNA purification. Genomic DNA was isolated and purified using a DNeasy Blood & Tissue Kit (Qiagen) in accordance with the manufacturer’s instructions. The quantity and purity of the genomic DNA were determined by the absorbance at 260 nm and the ratio of the absorbance at 260 and 280 nm (A260/280) using a NanoDrop ND-2000, respectively. As a result, we confirmed that A260/280 values of all samples were more than 1.7. The purified genomic DNAs were stored at −30 °C prior to use.

### Genome sequence analysis using Illumina MiSeq

Genome sequence analyses were performed with Illumina MiSeq System. A paired-end library was generated using a Nextera DNA Sample Prep Kit (Illumina) and was sequenced using a MiSeq Reagent Kit v3 (600-cycle) (Illumina) in accordance with the manufacturer’s instructions. We identified mutations by mapping paired-end reads to the MDS42 reference genome using the BRESEQ version 0.25 [45].

### Construction of deletion mutants

To construct deletion mutants of the *acrR*, *marR*, *ompF*, *glyXY*, and *atpI* genes, we deleted coding regions by Quick and Easy *E. coli* Gene Deletion Kit (Gene Bridges) in accordance with the manufacturer’s instructions. Briefly, to construct DNA fragments that had deleted coding regions, 60 bp homology arms were added to FRT-cm-FRT cassette (Gene Bridges), prokaryotic promoter and CP resistant gene flanked FRT sites, by sequential PCR. Each DNA fragment was purified by the MinElute PCR Purification Kit and then transformation to the parent strain and replacement of coding regions by FRT-cm-FRT cassette by λ phage recombinase were performed in accordance with the manufacturer’s instructions. After confirmation of replacement of coding regions by FRT-cm-FRT cassette, the CP resistant gene was excised by Flp-recombinase coded on 709-FLPe ap^R^ expression plasmid (Gene Bridges). After curing the expression plasmid, corresponding genomic regions were amplified by PCR and then confirmed by Sanger sequencing of the PCR products directly. *acrAB* genes, coding the multi-drug efflux pump and repressed by *acrR*, are located in the upper flanking region of *acrR*. To avoid the disruption of promoter and regulation regions of the *acrAB* operon, we deleted 118 bp of 3′ end of *acrR* including the stop codon.

## Additional files

Additional file 1: Figure S1. MICs of evolved strains obtained under single drug application. The MICs to (a) AMK, (b) ENX, and (c) CP of the parent strain (P) and strains evolved under single drug application (AMK, ENX, and CP) are presented. Evolved strains were obtained by isolating a single clone from the end-point culture of the laboratory evolution, and used to quantify MICs. (PDF 376 kb)

Additional file 2: Table S1. Mutations fixed in the evolved strains. The list was obtained by genome resequencing analysis using Illumina Miseq. (XLSX 28 kb)

## Abbreviations

AMK: Amikacin
ATP: Adenosine Triphosphate
CP: Chloramphenicol
ENX: Enoxacin
Ins/Del: Insertion and Deletion
IS: Insertion Sequence
MIC: Minimum inhibitory concentration
OD600: Optical Density at 600 nm
PCR: Polymerase Chain Reaction

## References

[1] Ho J, Tambyah PA, Paterson DL (2010). Multiresistant Gram-negative infections: a global perspective. Curr Opin Infect Dis.

[2] Levy SB, Marshall B (2004). Antibacterial resistance worldwide: causes, challenges and responses. Nat Med.

[3] Wang YC, Lipsitch M (2006). Upgrading antibiotic use within a class: tradeoff between resistance and treatment success. Proc Natl Acad Sci.

[4] Fischbach MA, Walsh CT (2009). Antibiotics for emerging pathogens. Science.

[5] Davies J, Davies D (2010). Origins and evolution of antibiotic resistance. Microbiol Mol Biol Rev.

[6] Norrby SR, Nord CE, Finch R. Lack of development of new antimicrobial drugs: a potential serious threat to public health. Lancet Infect Dis. 2005;5:115–9. Available from: https://www.ncbi.nlm.nih.gov/pubmed/15680781. 10.1016/S1473-3099(05)01283-1 15680781

[7] Bush K, Courvalin P, Dantas G, Davies J, Eisenstein B, Huovinen P (2011). Tackling antibiotic resistance. Nat Rev Microbiol.

[8] MacLean RC, Hall AR, Perron GG, Buckling A (2010). The population genetics of antibiotic resistance: integrating molecular mechanisms and treatment contexts. Nat Rev Genet.

[9] Yeh P, Tschumi AI, Kishony R (2006). Functional classification of drugs by properties of their pairwise interactions. Nat Genet.

[10] Michel J-B, Yeh PJ, Chait R, Moellering RC, Kishony R (2008). Drug interactions modulate the potential for evolution of resistance. Proc Natl Acad Sci U S A.

[11] Suzuki S, Horinouchi T, Furusawa C (2014). Prediction of antibiotic resistance by gene expression profiles. Nat Commun.

[12] Lázár V, Nagy I, Spohn R, Csörgő B, Györkei Á, Nyerges Á (2014). Genome-wide analysis captures the determinants of the antibiotic cross-resistance interaction network. Nat Commun.

[13] Imamovic L, Sommer MOA. Use of collateral sensitivity networks to design drug cycling protocols that avoid resistance development. Sci Transl Med. 2013;5:204ra132-204ra132. Available from: http://stm.sciencemag.org/content/5/204/204ra132%5Cn. https://www.ncbi.nlm.nih.gov/pubmed/24068739. 10.1126/scitranslmed.3006609 24068739

[14] Lázár V, Pal Singh G, Spohn R, Nagy I, Horváth B, Hrtyan M (2013). Bacterial evolution of antibiotic hypersensitivity. Mol Syst Biol.

[15] Rodriguez de Evgrafov M, Gumpert H, Munck C, Thomsen TT, Sommer MO (2015). Collateral Resistance and Sensitivity Modulate Evolution of High-Level Resistance to Drug Combination Treatment in Staphylococcus aureus. Mol Biol Evol.

[16] Suzuki S, Horinouchi T, Furusawa C (2015). Suppression of antibiotic resistance acquisition by combined use of antibiotics. J Biosci Bioeng.

[17] Kim S, Lieberman TD, Kishony R (2014). Alternating antibiotic treatments constrain evolutionary paths to multidrug resistance. Proc Natl Acad Sci U S A.

[18] Pena-Miller R, Laehnemann D, Jansen G, Fuentes-Hernandez A, Rosenstiel P, Schulenburg H, et al. When the Most Potent Combination of Antibiotics Selects for the Greatest Bacterial Load: The Smile-Frown Transition. Read AF, editor. PLoS Biol. 2013;11:e1001540. Public Library of Science. Available from: http://dx.plos.org/10.1371/journal.pbio.1001540. [cited 2017 Jan 20]. 10.1371/journal.pbio.1001540 PMC3635860 23630452

[19] Munck C, Gumpert HK, Wallin AI, Wang HH, Sommer MO (2014). Prediction of resistance development against drug combinations by collateral responses to component drugs. Sci Transl Med.

[20] Chait R, Craney A, Kishony R (2007). Antibiotic interactions that select against resistance. Nature.

[21] Hegreness M, Shoresh N, Damian D, Hartl D, Kishony R (2008). Accelerated evolution of resistance in multidrug environments. Proc Natl Acad Sci U S A.

[22] Gocke E (1991). Mechanism of quinolone mutagenicity in bacteria. Mutat Res.

[23] Ysern P, Clerch B, Castano M, Gibert I, Barde J, Llagostera M (1990). Induction of SOS genes in Escherichia coli and mutagenesis in Salmonella typhimurium by fluoroquinolones. Mutagenesis.

[24] Long H, Miller SF, Strauss C, Zhao C, Cheng L, Ye Z (2016). Antibiotic treatment enhances the genome-wide mutation rate of target cells. Proc Natl Acad Sci.

[25] Kohanski MA, DePristo MA, Collins JJ (2010). Sublethal antibiotic treatment leads to multidrug resistance via radical-induced mutagenesis. Mol Cell.

[26] Laureti L, Matic I, Gutierrez A (2013). Bacterial responses and genome instability induced by subinhibitory concentrations of antibiotics. Antibiot (Basel, Switzerland).

[27] Koo M-S, Lee J-H, Rah S-Y, Yeo W-S, Lee J-W, Lee K-L (2003). A reducing system of the superoxide sensor SoxR in Escherichia coli. EMBO J.

[28] Miller PF, Gambino LF, Sulavik MC, Gracheck SJ (1994). Genetic relationship between soxRS and mar loci in promoting multiple antibiotic resistance in Escherichia coli. Antimicrob Agents Chemother.

[29] Deka RK, Brautigam CA, Liu WZ, Tomchick DR, Norgard MV (2016). Molecular insights into the enzymatic diversity of flavin-trafficking protein (Ftp; formerly ApbE) in flavoprotein biogenesis in the bacterial periplasm. Microbiologyopen.

[30] Kanehisa M, Goto S, Hattori M, Aoki-Kinoshita KF, Itoh M, Kawashima S (2006). From genomics to chemical genomics: new developments in KEGG. Nucleic Acids Res.

[31] Shimizu K (2013). Metabolic regulation of a bacterial cell system with emphasis on Escherichia coli metabolism. ISRN Biochem.

[32] Mok WWK, Orman MA, Brynildsen MP (2015). Impacts of global transcriptional regulators on persister metabolism. Antimicrob Agents Chemother.

[33] Trindade S, Sousa A, Xavier KB, Dionisio F, Ferreira MG, Gordo I (2009). Positive epistasis drives the acquisition of multidrug resistance. PLoS Genet.

[34] Liu J, Hicks DB, Krulwich TA (2013). Roles of AtpI and two YidC-type proteins from alkaliphilic Bacillus pseudofirmus OF4 in ATP synthase assembly and nonfermentative growth. J Bacteriol.

[35] Wang H, Dzink-Fox JL, Chen M, Levy SB (2001). Genetic characterization of highly fluoroquinolone-resistant clinical Escherichia coli strains from China: role of acrR mutations. Antimicrob Agents Chemother.

[36] Delcour AH (2009). Outer membrane permeability and antibiotic resistance. Biochim Biophys Acta.

[37] Murakami S, Nakashima R, Yamashita E, Yamaguchi A (2002). Crystal structure of bacterial multidrug efflux transporter AcrB. Nature.

[38] Paulsen IT, Brown MH, Skurray RA (1996). Proton-dependent multidrug efflux systems. Microbiol Rev.

[39] Alekshun MN, Levy SB (1997). Regulation of chromosomally mediated multiple antibiotic resistance: the mar regulon. Antimicrob Agents Chemother.

[40] Okusu H, Ma D, Nikaido H (1996). AcrAB efflux pump plays a major role in the antibiotic resistance phenotype of Escherichia coli multiple-antibiotic-resistance (Mar) mutants. J Bacteriol.

[41] Weinreich DM, Lan Y, Wylie CS, Heckendorn RB (2013). Should evolutionary geneticists worry about higher-order epistasis?. Curr Opin Genet Dev..

[42] Wang HH, Isaacs FJ, Carr PA, Sun ZZ, Xu G, Forest CR, et al. Programming cells by multiplex genome engineering and accelerated evolution. Nature. 2009;460:894–8. Nature Publishing Group. Available from: http://www.nature.com/doifinder/10.1038/nature08187. [cited 2017 Feb 9]. 10.1038/nature08187 PMC4590770 19633652

[43] Mori E, Furusawa C, Kajihata S, Shirai T, Shimizu H (2011). Evaluating 13C enrichment data of free amino acids for precise metabolic flux analysis. Biotechnol J..

[44] Pósfai G, Plunkett G, Fehér T, Frisch D, Keil GM, Umenhoffer K, et al. Emergent properties of reduced-genome Escherichia coli. Science. 2006;312:1044–6. Available from: http://www.ncbi.nlm.nih.gov/pubmed/16645050. [cited 2011 Jul 18]. 10.1126/science.1126439 16645050

[45] Deatherage DE, Barrick JE. Identification of mutations in laboratory-evolved microbes from next-generation sequencing data using breseq. 2014. p. 165–88. Available from: http://link.springer.com/10.1007/978-1-4939-0554-6_12. [cited 2016 Jul 23]. 10.1007/978-1-4939-0554-6_12 PMC4239701 24838886

