# Supplementary figures and images for: Acceleration and suppression of resistance development by antibiotic combinations
Source: BMC Genomics. 2017 Apr 26;18:328. doi: 10.1186/s12864-017-3718-2 (PMC5405530; doi:10.1186/s12864-017-3718-2)

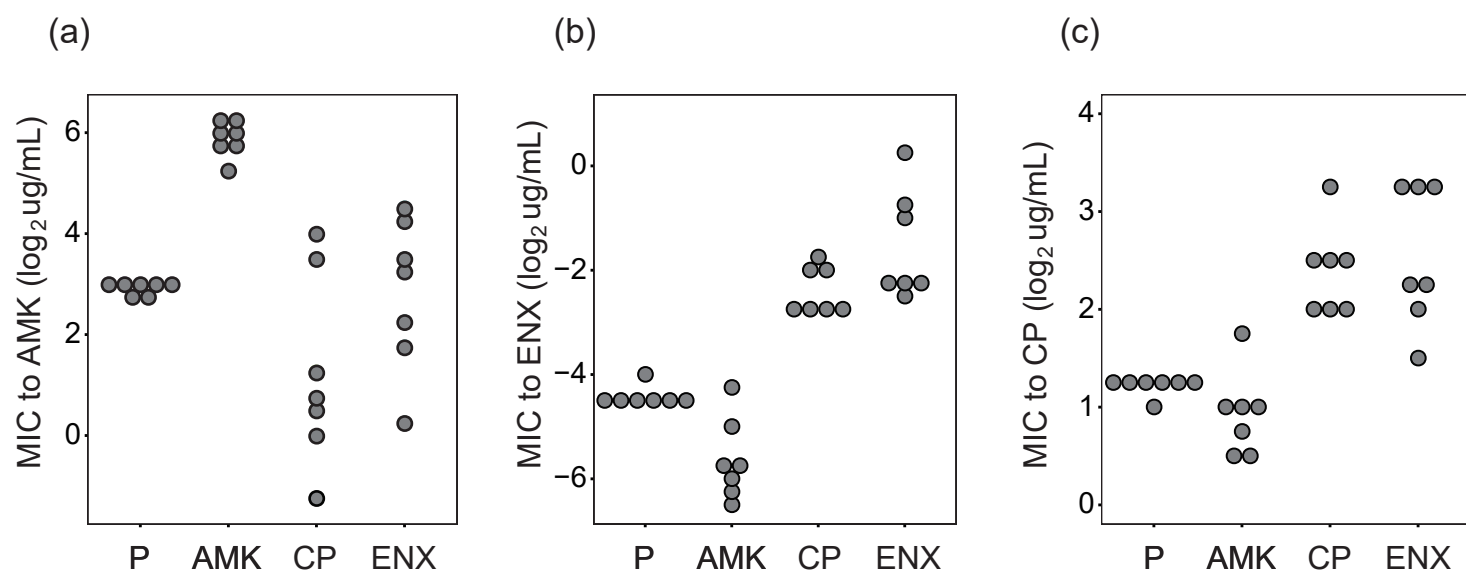

Suzuki, Horinouchi, and Furusawa, Figure S1

Supplement: Supplementary file 1 — MICs of evolved strains obtained under single drug application. The MICs to (a) AMK, (b) ENX, and (c) CP of the parent strain (P) and strains evolved under single drug application (AMK, ENX, and CP) are presented. Evolved strains were obtained by isolating a single clone from the end-point culture of the laboratory evolution, and used to quantify MICs. (PDF 376 kb) [file 12864_2017_3718_MOESM1_ESM.pdf]
